# Pathogenesis of plant-associated *Pseudomonas aeruginosa* in *Caenorhabditis elegans* model

**DOI:** 10.1186/s12866-022-02682-z

**Published:** 2022-11-09

**Authors:** Sakthivel Ambreetha, Dananjeyan Balachandar

**Affiliations:** Department of Agricultural Microbiology, Tamil Nadu Agricultural University, Coimbatore, 641003 Tamil Nadu India

**Keywords:** *Caenorhabditis elegans*, Plant-associated *Pseudomonas aeruginosa*, Pathogenicity, Pyocyanin, Virulence factors

## Abstract

**Background:**

*Pseudomonas aeruginosa* is a globally dreaded pathogen that triggers fatality in immuno-compromised individuals. The agricultural ecosystem is a massive reservoir of this bacterium, and several studies have recommended *P. aeruginosa* to promote plant growth. However, there were limited attempts to evaluate the health risks associated with plant-associated *P. aeruginosa*. The current study hypothesized that agricultural *P. aeruginosa* strains exhibit eukaryotic pathogenicity despite their plant-beneficial traits.

**Results:**

We have demonstrated that feeding with the plant-associated *P. aeruginosa* strains significantly affects *Caenorhabditis elegans* health. Out of the 18 *P. aeruginosa* strain tested, PPA03, PPA08, PPA10, PPA13, PPA14, PPA17, and PPA18 isolated from cucumber, tomato, eggplant, and chili exhibited higher virulence and pathogenicity. Correlation studies indicated that nearly 40% of mortality in *C. elegans* was triggered by the *P. aeruginosa* strains with high levels of pyocyanin (> 9 µg/ml) and biofilm to planktonic ratio (> 8).

**Conclusion:**

This study demonstrated that plant-associated *P. aeruginosa* could be a potential threat to human health similar to the clinical strains. Pyocyanin could be a potential biomarker to screen the pathogenic *P. aeruginosa* strains in the agricultural ecosystem.

## Background

*Pseudomonas aeruginosa* is an omnipresent bacterium commonly found in soil, water, moist surfaces, plants, animals, and humans. This bacterium is an opportunistic pathogen that causes terminal infections in patients with a weakened immune system. Its secondary metabolites, pyocyanin, rhamnolipid, and siderophores (pyochelin and pyoverdine), play a major role in establishing human infections [1]. In addition, biofilm formation is the key factor for *P. aeruginosa*-associated chronic obstructive lung infections [2]. Unfortunately, agricultural soil and plants have been the vast reservoirs of this bacterium [3]. Most of the *P. aeruginosa* strains in the agricultural systems are helpful for plant growth and protection [4–6]. However, some have also caused wilt and rot in the host plants [7, 8]. Despite multiple reports on the prevalence of *P. aeruginosa* in agricultural systems, there are limited studies on determining the associated health risks. Considering the opportunistic pathogenicity of this bacterium*,* it is crucial to examine the bio-safety of plant-associated *P. aeruginosa. Caenorhabditis elegans* is the standard model system used to determine the pathogenicity of clinical *P. aeruginosa* strains [9]. It is a bacterivorous nematode that can be cultured in the laboratory by supplying *Escherichia coli* OP50 as the food source. The *C. elegans* worms imitate the human innate immunity and rapidly exhibit sickness and death when fed virulent bacterial cells [10]. This model system can be used to determine if the ecological adaptation has reduced the virulence and pathogenicity of plant-associated *P. aeruginosa*. This might either validate the possible use of the plant-associated *P. aeruginosa* strains for agricultural sustainability or reveal their pathogenicity level and associated health hazards.

In our previous study, we isolated and characterized *P. aeruginosa* strains (PPA01-PPA18) from rhizospheric and endophytic niches of cucumber, tomato, eggplant, and chili harvested from different farms [11]. These strains had plant-beneficial traits such as mineral solubilization and plant-growth hormone production. In addition, all the strains inhibited the growth of bacterial and fungal phytopathogens, including *Xanthomonas oryzae*, *Pythium aphanidermatum*, *Rhizactonia solani*, and *Fusarium oxysporum* [12]. However, these plant-associated strains had several virulence traits, such as the production of pyocyanin, rhamnolipid, and siderophore, biofilm formation, swarming motility, and multiple lytic activities [11, 12]. In the current work, we hypothesized that one of these virulence factors could be used as a biomarker to detect the pathogenicity level of *P. aeruginosa* in agricultural systems*.* We tested if the variations in virulence factors can alter the impact of plant-associated *P. aeruginosa* on *C. elegans* survival*.* We have attempted to identify the critical virulence factor(s) contributing to the pathogenicity through this approach.

## Results

### *C. elegans* survival on plant-associated *Pseudomonas aeruginosa* strains

**Fig. 1:**
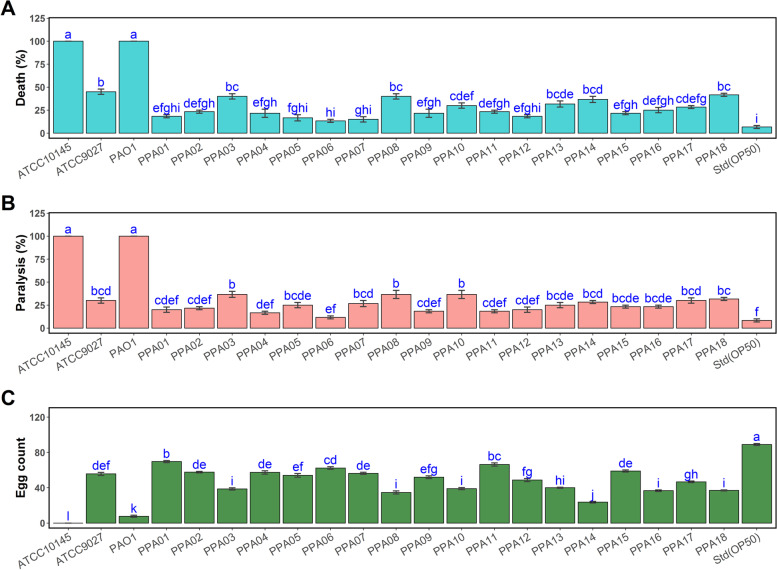
**A** Death, (**B**) paralysis, and (**C**) egg count of *C. elegans* as influenced by plant-associated and clinical strains of *P. aeruginosa*. ATCC10145, ATCC9027, and PAO1 – clinical strains; PPA01 to PPA18 – plant-associated strains; Std(OP50)—*E. coli* (OP50) standard nematode-feeding strain. Data represent the mean values (*n* = 3), and error bars indicate the standard error. For each panel, different letters indicate significant differences among the strains according to Tukey's test (α < 0.05)

The survival of *C. elegans* worms on feeding the plant-associated *P. aeruginosa* strains was predicted based on death, paralysis, and egg-laying (Fig. 1). Overall, three clinical *P. aeruginosa* strains (positive controls) caused significantly higher mortality (47—100%) when compared with the agricultural strains (12—42%) (Fig. 1A and B)*.* The reproductive health of the nematodes also drastically declined on feeding these strains (Fig. 1C). The nematodes fed with *E. coli* OP50 (negative control) exhibited the least death and paralysis, and their egg-laying ability remained unaffected (Fig. 1). All of the plant-associated *P. aeruginosa* strains caused a significantly higher negative impact on the nematode health when compared with the negative control (OP50). PPA03/cucumber; PPA08, PPA10/tomato; PPA13 and PPA14/eggplant; PPA17 and PPA18/chili were identified as the most pathogenic ones.

### Relative analyses of virulence factors and *C. elegans* pathogenicity in plant-associated ***P. aeruginosa*** strains

**Fig. 2:**
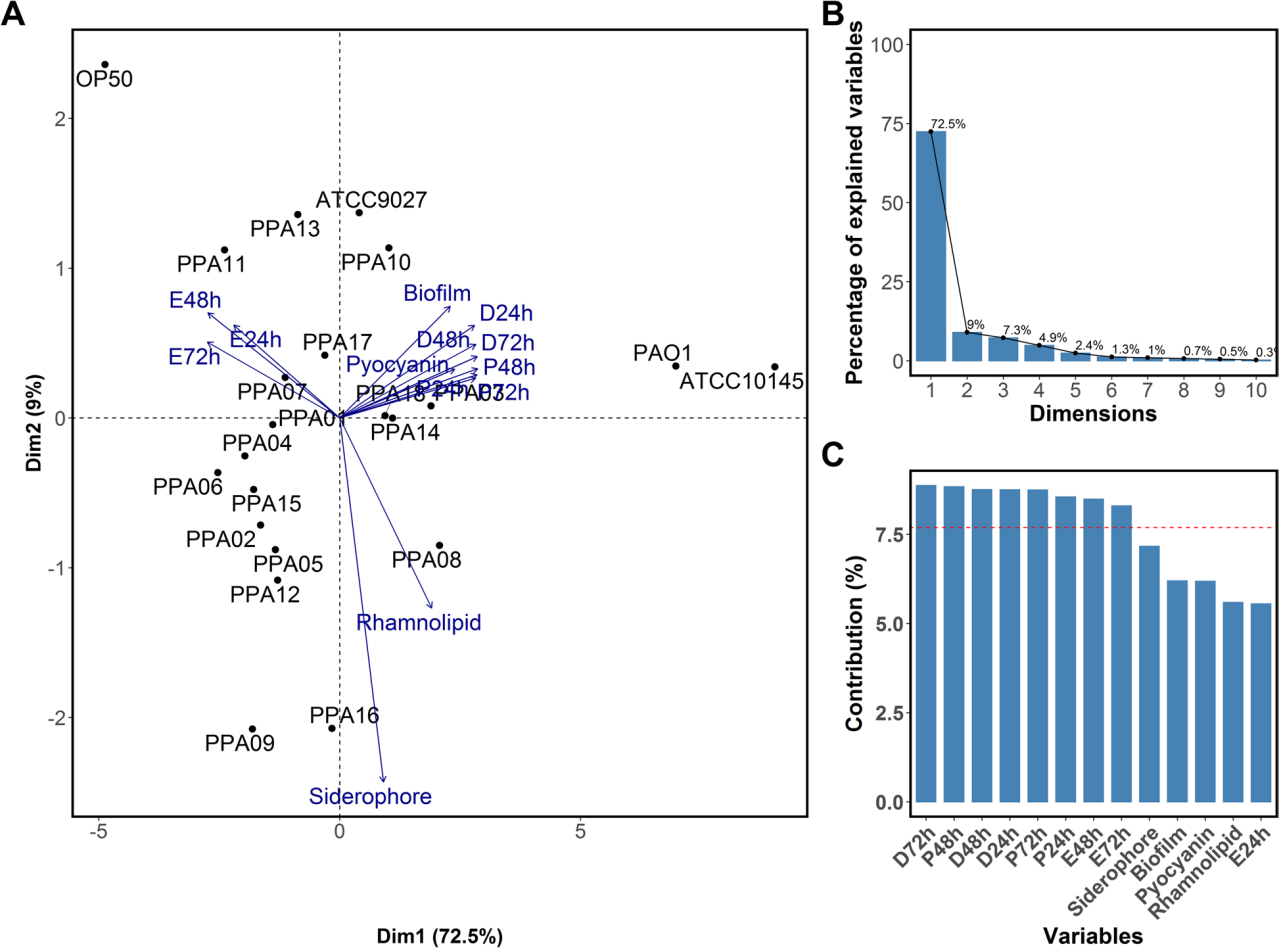
PCA relating virulence factors of plant-associated *P. aeruginosa* strains and their pathogenicity against *C. elegans*. **A** PCA biplot showing the position of each strain along with the orthogonal positions of the observed variables. The percentage variance explained by each principal component (Dim1 and Dim2) is given in parentheses in axes. **B** The percent contribution of each principal component to the cumulative variability in PCA. **C** The percent contribution of each variable on the axis identified by the principal component analysis. The red dotted line indicates significant loading values (> 0.70). D – death; P – paralysis; E – eggs at 24, 48, and 72 h incubation. ATCC10145, ATCC9027, and PAO1 – clinical strains; PPA01 to PPA18 – plant-associated strains; OP50—*E. coli* (OP50) standard nematode-feeding strain

Principal component analysis (PCA) identified the relation between plant-associated *P. aeruginosa* strains (PPA01-PPA18), their virulence factors (pyocyanin, rhamnolipid, siderophore, and biofilm), and *C. elegans* reproductive (egg-laying) and survival (paralysis and death) traits. The PCA biplot with two principal components (Dim1 and Dim2) depicted the orthogonal positions of the *P. aeruginosa* strains along with their virulence factors and pathogenicity against *C. elegans* (Fig. 2A). Dim1 and Dim2 contributed to 72.5% and 9% variability, respectively (Fig. 2B). Among the tested variables, the number of eggs laid by the *C. elegans* and paralytic and dead worms in 24, 48, and 72 h had high loading values (> 7.5) and significantly contributed to the Dim1 (Fig. 2C). The *P. aeruginosa* virulence factors majorly contributed to the Dim2. The *E. coli* OP50 (negative control) was positioned in the negative quadrant of the PCA plot while the clinical *P. aeruginosa* strains (positive controls) occupied the positive quadrant. Four plant-associated *P. aeruginosa* strains (PPA03/cucumber; PPA10/tomato; PPA14/eggplant; PPA18/chili) occupied the positive quadrant. The negative quadrant also had a few PPA strains (PPA07/tomato; PPA11, PPA13/eggplant; PPA17/chili).

### Segregating the plant-associated *P. aeruginosa* strains based on their pathogenicity against *C. elegans*

**Fig. 3:**
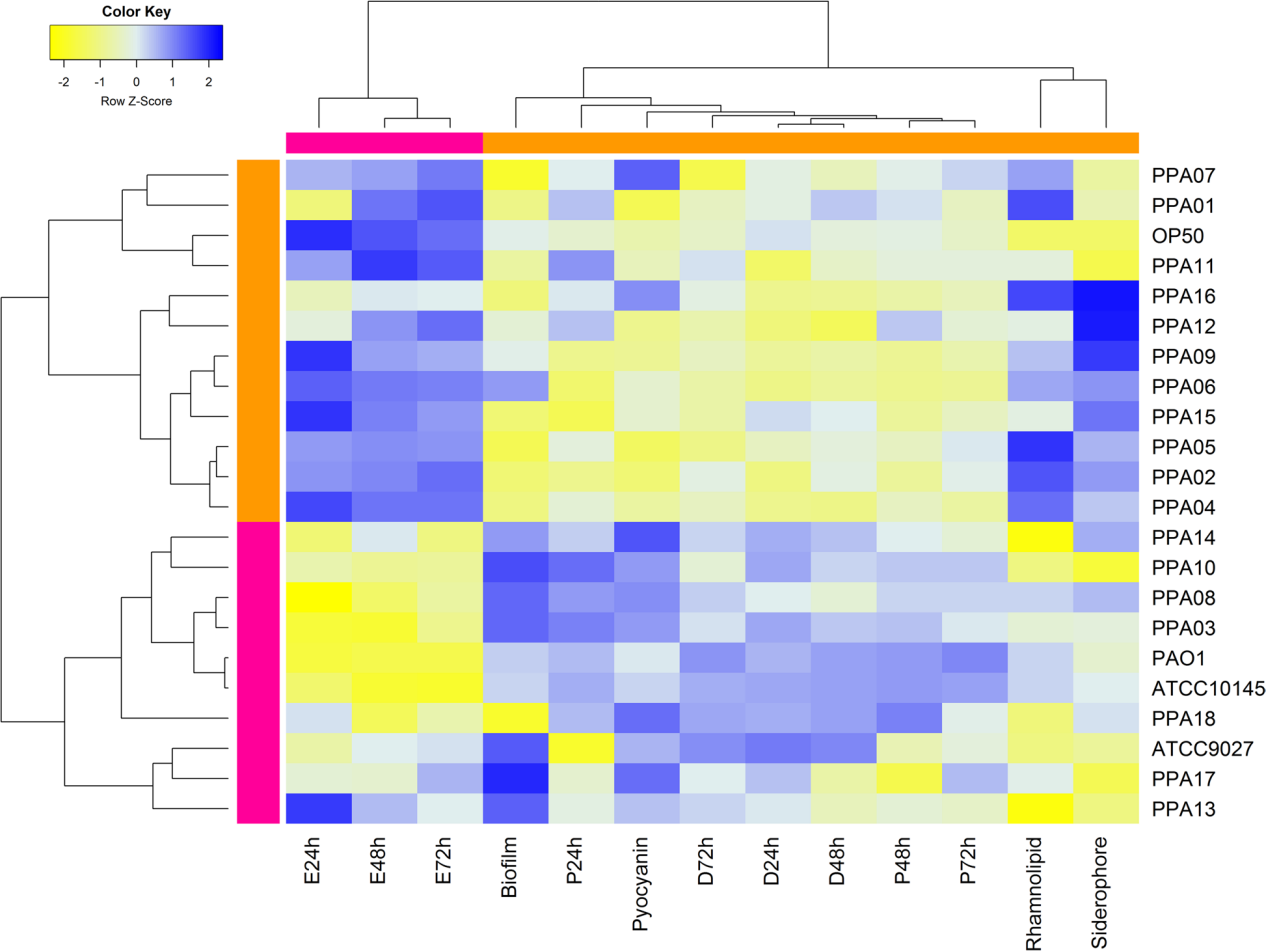
K-means clustering analysis of *P. aeruginosa* strains based on their pathogenicity against *C. elegans*. Double dendrogram and heatmap were created based on the K-means clustering and Spearman distance methods. The heatmap indicates the virulence and pathogenicity of the *P. aeruginosa* strains (yellow to blue – negative to positive). The top dendrogram reflects the clustering of the observed variables, while the left dendrogram indicates the grouping of the *P. aeruginosa* strains. D – death; P – paralysis; E – eggs at 24, 48, and 72 h incubation

K-means clustering analyses grouped the *P. aeruginosa* strains based on their impact on the reproduction and survival of the *C. elegans* nematodes (Fig. 3). Cluster A had the *E. coli* OP50 (negative control) along with 11 plant-associated *P. aeruginosa* strains (PPA01, PPA02, PPA04/cucumber; PPA05, PPA06, PPA07, PPA09/tomato; PPA11/eggplant; PPA15, PPA16/chili) that had low impact on the *C. elegans* survivability. The heatmap indicated that these strains had low virulence factors and led to minimal death and paralysis in *C. elegans*. The nematodes feeding on these strains laid a comparatively high number of eggs after 24, 48, and 72 h. Cluster B was occupied by the highly virulent clinical isolates of *P. aeruginosa* (PAO1, ATCC10145, and ATCC9027) along with eight plant-associated *P. aeruginosa* strains (PPA03/cucumber; PPA08, PPA10/tomato; PPA13, PPA14/eggplant; PPA17, PPA18/chili) that significantly affected the reproduction and survival of the *C. elegans.* These strains drastically reduced the number of eggs laid by the *C. elegans* (indicated by yellow) and led to a high number of paralytic- and dead worms (indicated by blue) after 24, 48, and 72 h of feeding. All the tested variables were clustered into two groups. The *P. aeruginosa* virulence factors and the number of paralytic- and dead worms clustered together. The number of eggs laid by the *C. elegans* from 24 to 72 h of feeding was grouped separately.

### Correlation between *P. aeruginosa* virulence factors and *C. elegans* survivability

**Fig. 4:**
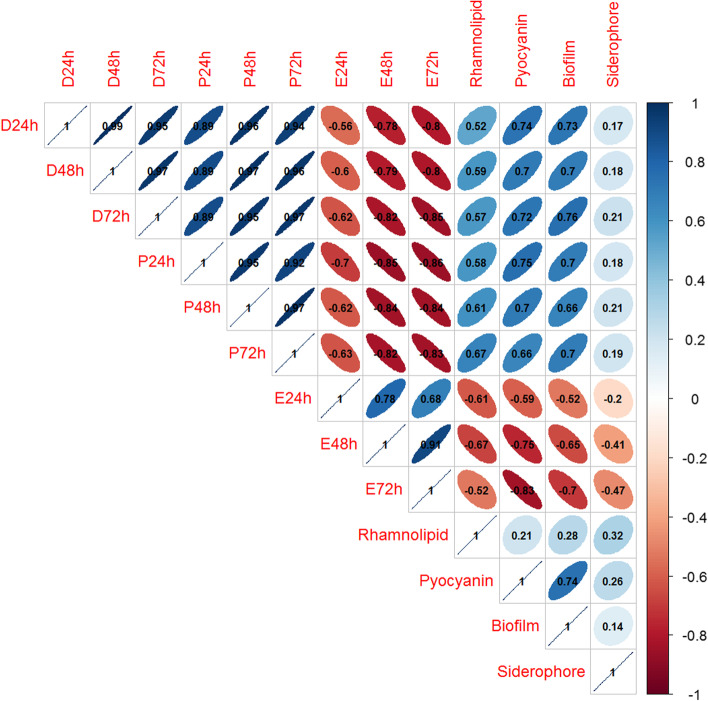
Pearson correlation plot for the *P. aeruginosa* virulence factors and *C. elegans* survival. Blue indicates positive correlation, and red indicates negative correlation. The level of correlation was further visualized through ellipses and their angles. D – death; P – paralysis; E – eggs at 24, 48, and 72 h incubation

Pearson correlation coefficient analyses determined the impact of each virulence factor produced by the plant-associated *P. aeruginosa* on the survivability of the *C. elegans* model (Fig. 4). The correlogram was created with a scale of -1 to 1 (red to blue). Among the four virulence traits tested, pyocyanin production and biofilm formation had the highest impact (> 0.65) on paralysis and death in *C. elegans* worms. The rhamnolipid had a moderate impact (> 0.5) on the *C. elgans* survival, while the siderophore exhibited the most negligible impact (< 0.25).

**Fig. 5:**
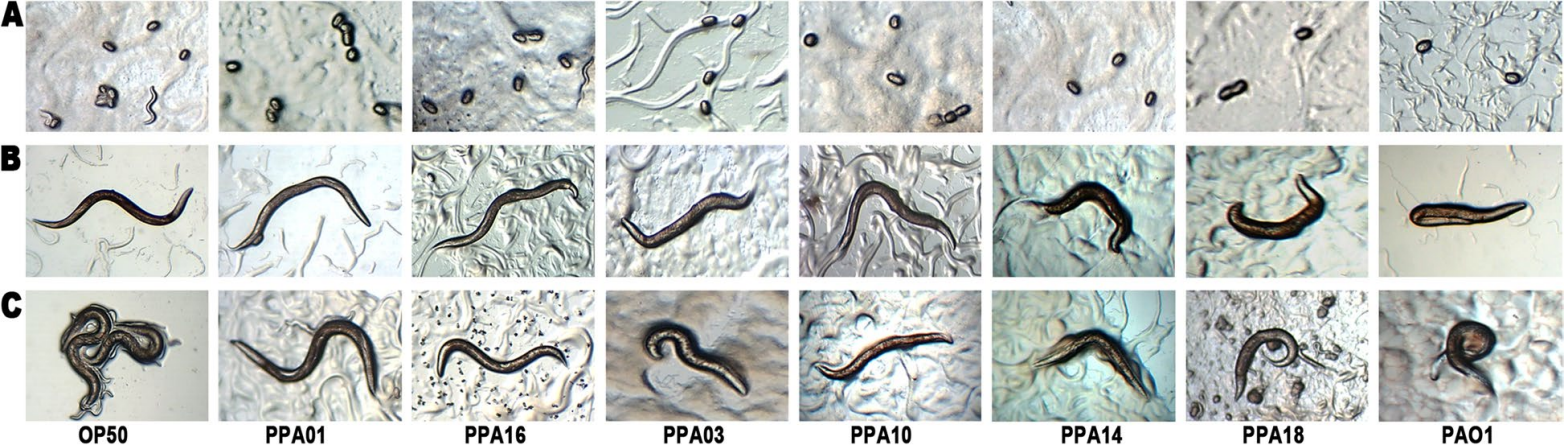
Impact of select strains of plant-associated *P. aeruginosa* on *C. elegans.* **A** Egg-laying, **B** Paralysis, and **C** Mortality. OP50 – negative control; *P. aeruginosa* PPA01/cucumber and PPA16/chili – high rhamnolipid producers with low levels of biofilm and pyocyanin; PPA03/cucumber and PPA10/tomato – high biofilm formers with low rhamnolipid and pyocyanin levels; PPA14/eggplant and PPA18/chili – high pyocyanin producers but low rhamnolipid and biofilm

The *P. aeruginosa* strains that produced high levels of pyocyanin (PPA14/eggplant; PPA18/chili) or biofilm (PPA03/cucumber; PPA10/tomato) caused high mortality and paralysis in *C. elegans* regardless of low rhamnolipid levels (Table 1; Fig. 5). On the contrary, the *P. aeruginosa* strains (PPA01/cucumber; PPA16/chili) with high rhamnolipid levels but less pyocyanin and biofilm caused relatively low paralysis and mortality in *C. elegans.* The clinical isolate, *P. aeruginosa* PAO1, which expressed high levels of all three virulence factors, triggered severe paralysis and death of the nematodes, while the *E. coli* OP50 (negative control) did not cause any harmful impact (Table 1; Fig. 5).

**Table 1:** Comparing the virulence levels and *C. elegans* pathogenicity of select strains of plant-associated *P. aeruginosa*

Source	Strain	Rhamnolipid	Pyocyanin	Biofilm	% of worms killed
Cucumber rhizosphere	PPA01	High	Low	Low	20
Chili endophyte	PPA16	High	Low	Low	26
Cucumber endophyte	PPA03	Low	Low	High	39
Tomato endophyte	PPA10	Low	Low	High	32
Eggplant rhizosphere	PPA14	Low	High	Low	36
Chili endophyte	PPA18	Low	High	Low	43
Positive control	PAO1	High	High	High	100
Negative control	OP50	Low	Low	Low	8

Rhamnolipid – High (> 14 µg/ml); Pyocyanin – High (> 9 µg/ml); Biofilm – High (> 8 Biofilm: planktonic population)

## Discussion

*Pseudomonas aeruginosa* is an opportunistic human pathogen that is omnipresent in multiple ecosystems. The strains that thrive in the agricultural system efficiently promote plant growth and inhibit phytopathogens [13, 14]. However, this bacterium is a Priority Level-I critical pathogen that causes terminal infections in immune-compromised individuals [15]. So far, there have been minimal works on the biosafety of plant-associated *P. aeruginosa* strains [16]. Our previous studies identified that agricultural *P. aeruginosa* strains harboring plant-beneficial traits could also exhibit virulence and pathogenicity [11, 12]. The present work identified the critical virulence factors that contribute to the eukaryotic pathogenesis of plant-associated *P. aeruginosa* using the *C. elegans* model system.

*C. elegans* is a standard model system used to determine the ability of clinical *P. aeruginosa* strains to cause mammalian infections [9, 17]. Several studies have demonstrated the pathogenicity of clinical *P. aeruginosa* strains using the *C. elegans* model [18–20]. However, the present work is the first attempt to use the *C. elegans* model to test the biosafety of plant-associated *P. aeruginosa*. *C. elegans* slow-killing is usually triggered due to the accumulation of pathogenic *P. aeruginosa* cells within the intestinal lumen of the nematodes [9]. In our study, the slow-killing assay revealed that plant-associated *P. aeruginosa* strains, PPA01-PPA18, exhibited relatively lower pathogenicity against *C. elegans* when compared to the clinical isolates, ATCC10145, ATCC9047, and PAO1. However, the most pathogenic PPA strains from eggplant (PPA14) and chili (PPA18) caused up to 43% of worm mortality.

Notably, these two strains produced more than 9 µg/ml of pyocyanin under in vitro conditions. Pyocyanin is a unique secondary metabolite produced by *P. aeruginosa.* It is a redox-active zwitterion that rapidly generates reactive oxygen species, leading to organ damage in the eukaryotic hosts [1, 21]. *P. aeruginosa* uses its pyocyanin to cause cytotoxic effects on respiratory, urological, vascular, and central nervous systems leading to multiple organ damage in the eukaryotic host [22]. A study on a highly pathogenic clinical strain, *P. aeruginosa* PA14, showed that three phenazine compounds such as 1-hydroxyphenazine, phenazine-1-carboxylic acid, and pyocyanin contribute to its pathogenicity [23]. In the current work, the plant-associated *P. aeruginosa* strains that produced high pyocyanin levels significantly affected *C. elegans* reproduction and survival (Fig. 5). Biofilm-forming *P. aeruginosa* causes chronic infections and increases the mortality rate in patients with critical pulmonary conditions [24–26]. The biofilm shields the *P. aeruginosa* cells from the host immune system and antibiotics, thereby facilitating persistent colonization [27]. In this study, we observed that high biofilm-formers could easily trigger *C. elegans* mortality. The plant-associated *P. aeruginosa* strains, PPA03 and PPA10, isolated from cucumber and tomato plants, respectively, had high biofilm levels and caused 30% death of the nematodes.

Rhamnolipid is one of the key virulence factors of *P. aeruginosa* that rupture the epithelial cells enabling the infiltration of mammalian lung tissues [28]. However, high rhamnolipid producing *P. aeruginosa* strains (PPA01/cucumber; PPA16/chili) caused relatively low death compared to the high pyocyanin producers. In the k-means clustering analyses, these strains occupied the low virulence group. Similarly, the siderophore production was less correlated with *C. elegans* paralysis and mortality than pyocyanin, rhamnolipid, and biofilm. However, pyoverdine-mediated hypoxia and death have previously been reported in the *C. elegans* fed with clinical *P. aeruginosa* [18].

Overall, seven out of these eighteen PPA strains (PPA03/cucumber; PPA08, and PPA10/tomato; PPA13, and PPA14/eggplant; PPA17, and PPA18/chili) tested in this study clustered together with the clinical strains based on their virulence and *C. elegans* pathogenicity (Fig. 2). This shows that the non-clinical *P. aeruginosa* strain could also be hazardous to human and animal health. Our study identified pyocyanin production and biofilm formation as the major pathogenicity determinants in the plant-associated *P. aeruginosa*. These two factors could be used as biomarkers to segregate the virulent and avirulent *P. aeruginosa* strains in the agricultural ecosystem.

## Conclusion

In conclusion, the plant-associated *P. aeruginosa* strains showed wide variation in their virulence factors which in turn alters their pathogenicity levels. Despite expressing comparatively lesser virulence than the clinical isolates, the plant-associated *P. aeruginosa* strains are pathogenic enough to cause paralysis and mortality in the *C. elegans* model*.* The *P. aeruginosa* strains in the agricultural ecosystem might evolve more pathogenic when exposed to the human and animal environment. Numerous studies have recommended *P. aeruginosa* strains to promote plant growth, alleviate abiotic stress, and protect plants against pests and insects [5, 8, 29, 30]. Based on our results, biosafety assessment is crucial before recommending an opportunistic bacterium for plant growth and protection. The risk associated with the agriculturally important *P. aeruginosa* strains can be detected based on their pyocyanin and biofilm levels. Such virulent and pathogenic *P. aeruginosa* strains in edible plants could cause potential health hazards to plants, animals, and humans with a weakened immune system.

## Materials and methods

### Strains and culture conditions

Plant-associated *P. aeruginosa* strains used in this study were previously isolated by the authors from cucumber, tomato, eggplant, and chili (Table 2; [11]). Clinical *P. aeruginosa* strains, PAO1, ATCC10145, and ATCC9027, were used as positive controls for pathogenicity assays [31–33]. These strains were grown at 37 °C in the *Pseudomonas* agar (for pyocyanin) medium (Himedia). *C. elegans* N2 hermaphrodite strain was cultured at 20 °C in the nematode growth medium (NGM) overlaid with *Escherichia coli* strain OP50 as a food source. The *E. coli* OP50 was periodically sub-cultured in the Luria Bertani (LB) medium and was used as a negative control for *C. elegans* reproduction and survival assays in all the experiments.

**Table 2:** Bacteria strains used in this study

Microorganism	Source^a^	Infection/Niche	References
**Plant-associated *P. aeruginosa* strains**			
PPA01	Cucumber	Rhizosphere	[11]
PPA02	Cucumber	Rhizosphere	[11]
PPA03	Cucumber	Endophyte	[11]
PPA04	Cucumber	Rhizosphere	[11]
PPA05	Tomato	Endophyte	[11]
PPA06	Tomato	Rhizosphere	[11]
PPA07	Tomato	Endophyte	[11]
PPA08	Tomato	Endophyte	[11]
PPA09	Tomato	Rhizosphere	[11]
PPA10	Tomato	Endophyte	[11]
PPA11	Eggplant	Endophyte	[11]
PPA12	Eggplant	Rhizosphere	[11]
PPA13	Eggplant	Rhizosphere	[11]
PPA14	Eggplant	Rhizosphere	[11]
PPA15	Chili	Rhizosphere	[11]
PPA16	Chili	Endophyte	[11]
PPA17	Chili	Endophyte	[11]
PPA18	Chili	Endophyte	[11]
***Pseudomonas aeruginosa* (reference strains)**			
PAO1	Human	Wound infection	[31]
ATCC9027	Human	External otitis	[32]
ATCC10145	Human	Unknown	[33]

^**a**^ The rhizospheric and endophytic niches of cucumber, tomato, eggplant, and chili cultivated in the orchards of Tamil Nadu Agricultural University, India (latitude, 11°07′3.36′′; longitude 76°59′39.91′′) was used for isolation of *Pseudomonas aeruginosa* strains

### Biofilm estimation

The *P. aeruginosa* cultures were grown in LB broth for 72 h, and the biofilm formation was estimated using the standard crystal violet-microtitre assay [34]. In brief, 25 µl of 24 h old cultures of the *P. aeruginosa* strains (OD660 ~ 0.5) were inoculated into 225 μl of LB broth in microtitre wells. After 72 h of incubation, A660 was measured (Spectramax® i3x, USA) to estimate the planktonic population. Biofilm attached to the microtitre wells was washed with sterile water and drenched with 300 µl of 0.1% crystal violet. After 10–15 min of incubation at room temperature, the plate was delicately washed with sterile water and allowed to dry for 24 h at room temperature. After 24 h, biofilm was dissolved using 30% acetic acid (300 µl), and absorbance was measured at 550 nm. Biofilm to the planktonic ratio (B:P) was determined for all the tested strains.

### Pyocyanin estimation

For pyocyanin assay, the cultures were grown in glycine-alanine broth for 48 h [35]. The pyocyanin was extracted from the cell-free supernatant using chloroform and spectrophotometrically quantified at 520 nm [36]. The A_520_ was multiplied with the pyocyanin extinction coefficient (17.072) to determine the concentration (µg/ml) [37].

### Rhamnolipid estimation

Rhamnolipid production was induced by growing the cells in protease peptone ammonium salts broth with a 2% (v/v) sunflower oil supplement [38]. Crude rhamnolipid was separated from the cell-free supernatant by chloroform–methanol extraction and quantified using the gravimetric method [39]. Briefly, the cell-free supernatant of 7 days old cultures was acidified with 12 M hydrochloric acid, and the rhamnolipid was extracted using a chloroform–methanol (2:1) mixture. The extracted lipids were concentrated, weighed, and expressed as µg/ml of the culture supernatant.

### Siderophore estimation

*P. aeruginosa* strains were grown overnight in succinate broth [40], and Chrome Azurol S (CAS)—shuttle assay was performed to quantify the total siderophore [41]. Briefly, an equal volume of CAS solution was added to the cell-free supernatant and incubated for an hour at ambient temperature. The absorbance was measured at 630 nm, and the percentage of siderophore was estimated based on the equation [(A_r_—A_s_)/A_r_] × 100, where A_r_ refers to the A_630_ of reference solution (mixture of CAS solution and uninoculated broth) and A_s_ refers to the A_630_ of the sample (mixture of CAS solution and culture supernatant) [40].

### *C. elegans* reproduction and survival assay

*C. elegans* gravid adults were ruptured using 1 N NaOH and 5% sodium hypochlorite (1:1) solution [42]. Their eggs were incubated in an M9 buffer for 24 h to allow hatching. L1-nematodes that emerged from these eggs were released into fresh OP50 lawns on NGM plates and allowed to grow up to the L4 stage. These L4-worms (20 per plate) were then released on NGM seeded with 50 µl of overnight grown *P. aeruginosa* strains (OD660 ~ 0.5) and were incubated at 20 °C [9, 10]. The reproductive ability of these worms was constantly monitored based on the number of eggs laid after 24, 48, and 72 h of feeding. The impact of *P. aeruginosa* strains on *C. elegans* survival was estimated based on paralysis and death. The worms were scored paralytic when they turned non-motile post-feeding. The nematodes that did not respond to physical stimulus were considered dead. The paralytic and dead worms were counted at 24, 48, and 72 h of incubation. All the experiments were performed with three replicates.

### Statistical analyses

The statistical analyses were performed in R software (Version 4.1.1) (R Core Team, Vienna, Austria). The *C. elegans* pathogenicity data were tested with a one-way analysis of variance (ANOVA) followed by Tukey's honestly significant difference test at α = 0.05. The PCA was performed for all the assessed variables using the princomp function of the factoextra-package of R. The PCA biplot, contribution plot, and eigenvalues corresponding to the variation explained by each principal component were visualized using the fviz function of factoextra. K-means clustering heatmap was generated using the heatmap.2 R package to group the PPA strains based on their virulence factors and *C. elegans* survival. The correlation between the assessed variables was evaluated based on Pearson's correlation and visualized through Corrplot-package.

## Data Availability

The datasets generated and/or analyzed during the current study are available from the corresponding author on reasonable request.

## Abbreviations

PPA: Plant-associated Pseudomonas aeruginosa
PCA: Principal component analysis
DIM: Dimension
LB: Luria Bertani
B:P: Biofilm:Planktonic
CAS: Chrome-Azural S
ANOVA: Analysis of variance

## References

[1] Moradali MF, Ghods S, Rehm BHA (2017). *Pseudomonas aeruginosa* lifestyle: A paradigm for adaptation, survival, and persistence. Front Cell Infect Microbiol.

[2] Mulcahy LR, Isabella VM, Lewis K (2014). *Pseudomonas aeruginosa* biofilms in disease. Microb Ecol.

[3] Green SK, Schroth MN, Cho JJ, Kominos SD, Vitanza-Jack VB (1974). Agricultural plants and soil as a reservoir for *Pseudomonas aeruginosa*. Appl Microbiol.

[4] Gupta V, Buch A (2019). *Pseudomonas aeruginosa* predominates as multifaceted rhizospheric bacteria with combined abilities of P-solubilization and biocontrol. J Pure Appl Microbiol.

[5] Sancheti A, Ju L-K (2019). Eco-friendly rhamnolipid based fungicides for protection of soybeans from *Phytophthora sojae*. Pest Manag Sci.

[6] Chandra H, Kumari P, Bisht R, Prasad R, Yadav S (2020). Plant growth promoting *Pseudomonas aeruginosa* from *Valeriana Wallichii* displays antagonistic potential against three phytopathogenic fungi. Mol Biol Rep.

[7] Gao J, Wang Y, Wang CW, Lu BH (2014). First report of bacterial root rot of ginseng caused by *Pseudomonas aeruginosa* in China. Plant Dis.

[8] Tiwari P, Singh JS (2017). A plant growth promoting rhizospheric *Pseudomonas aeruginosa* strain inhibits seed germination in *Triticum aestivum* (L) and *Zea mays* (L). Microbiol Res.

[9] Tan M-W, Mahajan-Miklos S, Ausubel FM (1999). Killing of *Caenorhabditis elegans* by *Pseudomonas aeruginosa* used to model mammalian bacterial pathogenesis. PNAS.

[10] Adonizio A, Kong K-F, Mathee K (2008). Inhibition of quorum sensing-controlled virulence factor production in *Pseudomonas aeruginosa* by South Florida plant extracts. Antimicrob Agents Chemother.

[11] Ambreetha S, Marimuthu P, Mathee K, Balachandar D (2021). Rhizospheric and endophytic *Pseudomonas aeruginosa* in edible vegetable plants share molecular and metabolic traits with clinical isolates. J Appl Microbiol.

[12] Ambreetha S, Marimuthu P, Mathee K, Balachandar D (2022). Plant-associated *Pseudomonas aeruginosa* strains harbor multiple virulence traits critical for human infection. J Med Microbiol.

[13] Ahemad M, Khan MS (2010). Phosphate-solubilizing and plant-growth-promoting *Pseudomonas aeruginosa* PS1 improves greengram performance in quizalafop-p-ethyl and clodinafop amended soil. Arch Environ Contam Toxicol.

[14] Sun X, Xu Y, Chen L, Jin X, Ni H (2021). The salt-tolerant phenazine-1-carboxamide-producing bacterium *Pseudomonas aeruginosa* NF011 isolated from wheat rhizosphere soil in dry farmland with antagonism against *Fusarium graminearum*. Microbiol Res.

[15] TalebiBezminAbadi A, Rizvanov AA, Haertlé T, Blatt NL (2019). World Health Organization report: Current crisis of antibiotic resistance. BioNanoScience..

[16] Kumar A, Munder A, Aravind R, Eapen SJ, Tümmler B, Raaijmakers JM (2013). Friend or foe: genetic and functional characterization of plant endophytic *Pseudomonas aeruginosa*. Environ Microbiol.

[17] Tan MW, Rahme LG, Sternberg JA, Tompkins RG, Ausubel FM (1999). Pseudomonas aeruginosa killing of Caenorhabditis elegans used to identify P. aeruginosa virulence factors. PNAS.

[18] Kirienko NV, Cezairliyan BO, Ausubel FM, Powell JR, Filloux A, Ramos JL (2014). *Pseudomonas aeruginosa* PA14 pathogenesis in *Caenorhabditis elegans*. *Pseudomonas Methods and Protocols*.

[19] Kirienko NV, Kirienko DR, Larkins-Ford J, Wählby C, Ruvkun G, Ausubel FM (2013). *Pseudomonas aeruginosa* disrupts *Caenorhabditis elegans* iron homeostasis, causing a hypoxic response and death. Cell Host Microbe.

[20] Gallagher LA, Manoil C (2001). *Pseudomonas aeruginosa* PAO1 kills *Caenorhabditis elegans* by cyanide poisoning. J Bacteriol.

[21] Moayedi A, Nowroozi J, Akhavan Sepahy A (2018). Cytotoxic effect of pyocyanin on human pancreatic cancer cell line (Panc-1). Iran J Basic Med Sci.

[22] Hall S, McDermott C, Anoopkumar-Dukie S, McFarland AJ, Forbes A, Perkins AV, Davey AK, Chess-Williams R, Kiefel MJ, Arora D (2016). Cellular effects of pyocyanin, a secreted virulence factor of *Pseudomonas aeruginosa*. Toxins.

[23] Cezairliyan B, Vinayavekhin N, Grenfell-Lee D, Yuen GJ, Saghatelian A, Ausubel FM (2013). Identification of *Pseudomonas aeruginosa* phenazines that kill *Caenorhabditis elegans*. PLoS Pathog.

[24] Nixon GM, Armstrong DS, Carzino R, Carlin JB, Olinsky A, Robertson CF, Grimwood K (2001). Clinical outcome after early *Pseudomonas aeruginosa* infection in cystic fibrosis. J Pediatr.

[25] Bjarnsholt T, Jensen PO, Fiandaca MJ, Pedersen J, Hansen CR, Andersen CB, Pressler T, Givskov M, Høiby N (2009). *Pseudomonas aeruginosa* biofilms in the respiratory tract of cystic fibrosis patients. Pediatr Pulmonol.

[26] Singh PK, Schaefer AL, Parsek MR, Moninger TO, Welsh MJ, Greenberg EP (2000). Quorum-sensing signals indicate that cystic fibrosis lungs are infected with bacterial biofilms. Nature.

[27] Abdulhaq N, Nawaz Z, Zahoor MA, Siddique AB (2020). Association of biofilm formation with multi drug resistance in clinical isolates of *Pseudomonas aeruginosa*. EXCLI J.

[28] Zulianello L, Canard C, Köhler T, Caille D, Lacroix J-S, Meda P (2006). Rhamnolipids are virulence factors that promote early infiltration of primary human airway epithelia by *Pseudomonas aeruginosa*. Infect Immun.

[29] Roychowdhury R, Qaiser TF, Mukherjee P, Roy M (2019). Isolation and characterization of a *Pseudomonas aeruginosa* strain PGP for plant growth promotion. Proc Natl Acad Sci India, Sect B Biol Sci.

[30] Monnier N, Furlan A, Botcazon C, Dahi A, Mongelard G, Cordelier S, Clément C, Dorey S, Sarazin C, Rippa S. Rhamnolipids from *Pseudomonas aeruginosa* are elicitors triggering *Brassica napus* protection against *Botrytis cinerea* without physiological disorders. Front Plant Sci. 2018;9:1170. 10.3389/fpls.2018.01170 PMC6092566 30135699

[31] Holloway BW (1955). Genetic recombination in *Pseudomonas aeruginosa*. J Gen Microbiol.

[32] Haynes WC (1951). Pseudomonas aeruginosa–-its characterization and identification. Microbiol.

[33] Picard B, Denamur E, Barakat A, Elion J, Goullet P (1994). Genetic heterogeneity of *Pseudomonas aeruginosa* clinical isolates revealed by esterase electrophoretic polymorphism and restriction fragment length polymorphism of the ribosomal RNA gene region. J Med Microbiol.

[34] O'Toole GA (2011). Microtiter dish biofilm formation assay. J Viz Exp.

[35] Ingledew WM, Campbell JJ (1969). A new resuspension medium for pyocyanine production. Can J Microbiol.

[36] Essar DW, Eberly L, Hadero A, Crawford IP (1990). Identification and characterization of genes for a second anthranilate synthase in *Pseudomonas aeruginosa*: interchangeability of the two anthranilate synthases and evolutionary implications. J Bacteriol.

[37] Kurachi M (1958). Studies on the biosynthesis of pyocyanine (II): Isolation and determination of pyocyanine. Bulletin of the Institute for Chemical Research, Kyoto University.

[38] Zhang Y, Miller RM (1992). Enhanced octadecane dispersion and biodegradation by a *Pseudomonas* rhamnolipid surfactant (biosurfactant). Appl Environ Microbiol.

[39] Gunther NW, Nunez A, Fett W, Solaiman DK (2005). Production of rhamnolipids by *Pseudomonas chlororaphis*, a nonpathogenic bacterium. Appl Environ Microbiol.

[40] Pérez-Miranda S, Cabirol N, George-Téllez R, Zamudio-Rivera LS, Fernández FJ (2007). O-CAS, a fast and universal method for siderophore detection. J Microbiol Methods.

[41] Schwyn B, Neilands JB (1987). Universal chemical assay for the detection and determination of siderophores. Anal Biochem.

[42] Brenner S (1974). The genetics of *Caenorhabditis elegans*. Genetics.

